# A new, long-term daily satellite-based rainfall dataset for operational monitoring in Africa

**DOI:** 10.1038/sdata.2017.63

**Published:** 2017-05-23

**Authors:** Ross I. Maidment, David Grimes, Emily Black, Elena Tarnavsky, Matthew Young, Helen Greatrex, Richard P. Allan, Thorwald Stein, Edson Nkonde, Samuel Senkunda, Edgar Misael Uribe Alcántara

**Affiliations:** 1 Department of Meteorology, University of Reading, Reading RG6 6BB, UK; 2 International Research Institute for Climate and Society (IRI), Columbia University, New York, NY 10964-1000, USA; 3 National Centre for Earth Observation (NCEO), Reading RG6 6BB, UK; 4 Zambian Meteorological Department, P.O. Box 30200, Lusaka, Zambia; 5 Uganda National Meteorological Authority, P.O. Box 7025, Kampala, Uganda; 6 Universidad Autonoma del Estado de Hidalgo, Pachuca 42039, Mexico

**Keywords:** Natural hazards, Climate sciences, Hydrology

## Abstract

Rainfall information is essential for many applications in developing countries, and yet, continually updated information at fine temporal and spatial scales is lacking. In Africa, rainfall monitoring is particularly important given the close relationship between climate and livelihoods. To address this information gap, this paper describes two versions (v2.0 and v3.0) of the TAMSAT daily rainfall dataset based on high-resolution thermal-infrared observations, available from 1983 to the present. The datasets are based on the disaggregation of 10-day (v2.0) and 5-day (v3.0) total TAMSAT rainfall estimates to a daily time-step using daily cold cloud duration. This approach provides temporally consistent historic and near-real time daily rainfall information for all of Africa. The estimates have been evaluated using ground-based observations from five countries with contrasting rainfall climates (Mozambique, Niger, Nigeria, Uganda, and Zambia) and compared to other satellite-based rainfall estimates. The results indicate that both versions of the TAMSAT daily estimates reliably detects rainy days, but have less skill in capturing rainfall amount—results that are comparable to the other datasets.

## Background and Summary

High spatial and temporal rainfall variability is a major challenge when it comes to managing agricultural activities across Africa, as above or below average rainfall can lead to crop losses and failure^1^. A notable recent example was the occurrence of widespread drought conditions across the Horn of Africa during 2010–2011 which affected over 10 million people^2,3^. To help mitigate these climate-related risks, access to reliable rainfall information, both historic and near-real time, is a necessity. Historic data allows climate risks (e.g., the probability of drought) and long-term changes in the rainfall climate to be assessed, while near-real time data is important to evaluate the present day weather in a historical context. The latter is especially important in monitoring the evolution of hydrological hazards, allowing timely responses from governments and organizations before major crises occurs. Although temporally coarse data (for example, dekadal or monthly) can be useful for evaluating climatic trends and monitoring above or below average rainfall^4^, information at fine time scales (e.g., daily) provides information valuable in a range of other applications such as crop modelling, water management and weather index-based insurance^4,5^.

Conventionally, rain gauge records provide the most accurate means to obtain information about the rainfall climate. However, the spatially sparse network and often temporally incomplete records at many stations across Africa leaves large parts of the continent unobserved^6^. This problem is exacerbated by the high spatial variability associated with convective rainfall at the daily time-step that makes a rain gauge measurement only representative of rainfall over several square kilometres surrounding the gauge^7^. Except in the vicinity of a continually reporting weather station, gauge observations alone are impractical for the routine assessment of rainfall. Africa-wide, near-real time gauge records are only available via the Global Telecommunications System (GTS) network, usually through automatic weather stations. Although over 700 stations are registered on the GTS network, only a small proportion of these report daily^6,8^. Moreover, access to country-level records that often contain more data than is publicly available, is often only possible via direct contact with African meteorological and hydrological agencies.

The limitations associated with gauge measurements have elevated the importance of satellite-based rainfall estimates in many applications across Africa, especially in agriculture and drought monitoring^8^. Satellite-based algorithms have the advantage of providing full spatial coverage and have been demonstrated to be skilful in many locations over Africa^9–17^. While there is an ever growing collection of satellite-based datasets capable of providing near-real time estimates (a selection of which are listed in Table 1 in Maidment *et al.*^18^), only a handful of publicly available high resolution satellite-based datasets providing historic data (at least 30 years) at the daily time-step and which are continually updated in real time or near-real time, exist for Africa. These are the National Oceanic Atmospheric Administration (NOAA) African Rainfall Climatology version 2.0 (ARC^19^) and the Climate Hazards Group InfraRed Precipitation with Station data version 2.0 (CHIRPS^20^) and are described in the Technical Validation section. Given the dearth of Africa-wide long-term (30 years or more) daily rainfall information and large uncertainties in existing observational records over Africa^21–23^, the addition of daily satellite-based rainfall datasets with contrasting estimation approaches are extremely valuable for rainfall monitoring and climate research. Moreover, Africa’s population is expanding rapidly and it is expected that this trend will continue throughout this century^24^. The pressures such growth is putting on agricultural and water resources, combined with changes in the rainfall climate^21^, are encouraging the use of climate-based services such as Enhancing National Climate Services (ENACTS)^25^ and Rainwatch^26^ in many African countries. These services provide easily accessible historic and near-real time information on the local climate that is useful to a wide range of stakeholders. Such platforms, however, require skilful, long-term and regularly updated rainfall information.

Here, we describe and evaluate two versions (2.0 and 3.0) of the long-term daily TAMSAT (Tropical Applications of Meteorology using SATellite and ground based observations) rainfall dataset (Data Citation 1 and Data Citation 2; hereinafter TAMSAT-2 and TAMSAT-3 respectively), based on high resolution Meteosat thermal-infrared (TIR) observations for all of Africa, available from 1983 to the present and updated in near-real time. TAMSAT-2 and TAMSAT-3 are based on the disaggregation of the TAMSAT version 2.0 dekadal^18^ and TAMSAT version 3.0 pentadal rainfall estimates respectively, to a daily time-step using daily calibrated cold cloud duration (CCD) observations (see Methods section for algorithm details).

In January 2017, the TAMSAT Group released TAMSAT version 3.0—which is produced operationally alongside version 2.0 (ref. 18). Given that the daily rainfall estimates derived from TAMSAT v2.0 have been in the public domain for several years and are used by many users, this paper formally evaluates both TAMSAT-2 and TAMSAT-3. The rainfall estimates have been validated using daily rain gauge measurements from five Africa countries (Mozambique, Niger, Nigeria, Uganda, and Zambia) and compared with estimates from six other satellite-based rainfall datasets, some of which are used widely in rainfall monitoring applications across Africa.

## Methods

### TAMSAT algorithm

The daily estimates (both TAMSAT-2 and TAMSAT-3) are derived from the TAMSAT rainfall estimation algorithm. The TAMSAT Group have, since the 1980s, produced estimates at the 10-day (dekad) scale. The algorithm, described in Milford *et al.*^27^, Dugdale *et al.*^28^, Grimes *et al.*^10^ and Maidment *et al.*^18^, works on the premise that the use of TIR imagery to monitor the cold cloud tops of rain-bearing convective cumulonimbus systems acts as a useful indicator for rainfall in the Tropics. Despite the simplicity of the TAMSAT operational approach, the dekadal estimates have been shown to perform well where rainfall is predominantly convective in origin^9,12,14,29–32^. The TAMSAT-2 estimates, described in this paper, have also been evaluated over the complex terrain of Ethiopia and demonstrated good skill^33^. Such skill, both at the daily and dekadal time-step, underlines the effectiveness of using TIR imagery in rainfall estimation where and when rainfall is convective in origin. The TAMSAT approach to rainfall estimation, however, does have limitations. Where rainfall from warm rain processes is dominant, such as along the coastal parts of West Africa^34^ and over mountainous regions^31^, the ability to identify rainy cloud is reduced. In addition, since the TAMSAT estimation approach is geared towards drought monitoring where accurately representing low rainfall totals is important, the algorithm in TAMSAT v2.0 (and all previous versions) was calibrated to better capture the more frequent, low rainfall amounts^18^. In doing so, the total rainfall is underestimated, resulting in an inherent dry bias that is more pronounced when the data (both daily and dekadal estimates) are aggregated (in space and/or time).

The aforementioned dry bias in TAMSAT v2.0 dekadal data, along with unrealistic spatial artefacts that originated from the use of rectangular calibration zones, prompted the TAMSAT Group to modify the calibration design, while ensuring the data is still applicable to drought monitoring. Although the principle features of the TAMSAT rainfall estimation approach have remained the same, the calibration used in version 3.0 differs markedly to version 2.0 and is designed to better capture local variations in the rainfall climate while reducing problems associated with version 2.0. Additionally, the time-step for the primary rainfall estimate is now 5-day (pentad), compared to 10-day in version 2.0. Here, we provide an outline of the common features behind the methodology used to create both TAMSAT-2 and TAMSAT-3. Comprehensive details on the version 2.0 pan-African calibration can be found in Maidment *et al.*^18^ and Tarnavsky *et al.*^8^, while details on version 3.0 can be found on the TAMSAT website (http://www.tamsat.org.uk).

**Figure 1:**
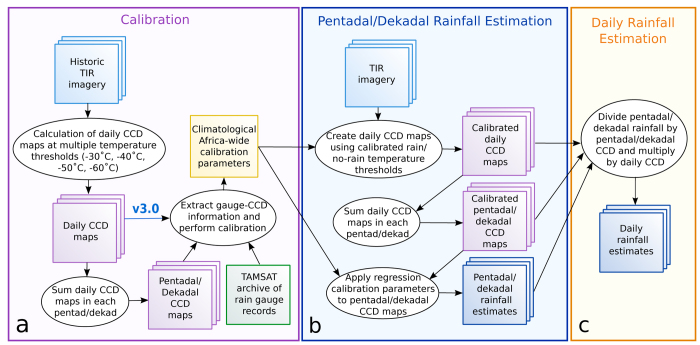
Schematic summarising the TAMSAT daily rainfall estimation process. (**a**) calibration of the algorithm at the dekadal (version 2.0) and pentadal (version 3.0) time-step, (**b**) estimation of the pentadal/dekadal rainfall estimates and (**c**) estimation of the daily rainfall estimates. Squares denote inputs or outputs and ovals denote processes. Note that the process is identical for versions 2.0 and 3.0 except where indicated on the diagram.

The TAMSAT algorithm is based on two primary data inputs: Meteosat TIR imagery provided by The European Organisation for the Exploitation of Meteorological Satellites (EUMETSAT) and rain gauge observations for calibration (see Fig. 1 for the estimation process). The rainfall estimation approach is based on TIR imagery obtained every 15 min from July 2006 and every 30 min prior to this. The TAMSAT algorithm is an example of a cloud-indexing method: the duration of cloud tops exceeding a predetermined temperature threshold, known as cold cloud duration (CCD), acts as a proxy for rainfall.

The calibration process is divided into two stages. The first stage distinguishes rainy regions from non-rainy regions, while the second stage attempts to assign a rainfall amount for the rainy regions. In the first stage, daily CCD totals are derived at a range of thresholds between −30 °C and −60 °C. These are then summed to the dekadal (in v2.0) or pentadal (in v3.0) time-step and a set of contingency tables are prepared for every threshold, comparing greater than zero CCD at the pixel scale with rainfall occurrence from the collocated rain gauge records. The temperature threshold with the greatest skill for determining rainfall events (greater than 0 mm) is selected based primarily on the rainfall event frequency bias (see Maidment *et al.*^18^ for details). In version 2.0, these were determined for large climatologically-similar rectangular zones, whereas in version 3.0, these are derived over 1.0° grid boxes (hence capturing local detail more accurately) where sufficient gauges exist and then interpolated Africa-wide. In the second stage, calibration parameters are obtained by linearly regressing CCD totals for the selected temperature threshold with historical rain gauge accumulations. In version 3.0, a spatially and temporally varying bias adjustment is then made to the calibration parameters. Using the calibration coefficients, rainfall is estimated as a function of CCD, according to equation (1):

(1)raintimestep={a0+a1CCDtimestepCCD>00CCD=0

Where *timestep* is either pentad or dekad, depending on the TAMSAT version, and *a*_0_ and *a*_1_ are the linear calibration coefficients. If CCD is equal to zero, rainfall is also assumed to be zero. The TAMSAT method implements a local calibration, hence the linear calibration coefficients vary spatially and monthly to reflect the geographical and temporal variations in the average rainfall climate across Africa^8^.

The TAMSAT-2 data are derived from TAMSAT dekadal v2.0 estimates that constitute the TAMSAT African Rainfall Climatology And Time-series (TARCAT) dataset^18^ which is still routinely updated to the present day. Since the calibrations used in these datasets do not change from year-to-year, the interannual variations in rainfall are dependent only on the satellite observations. The TAMSAT method thus contrasts with other long-term datasets such as CHIRPS and ARC, which merge gauge data in near-real time^19,20^. The inclusion of contemporaneous gauge data arguably makes maximal use of all available data sources, increasing skill where high quality gauge data are available. The African gauge network is, however, not consistent in either time or space, and the inclusion of gauge data may thus introduce artefacts, especially when assessing long term change^21^. The TAMSAT datasets hence can be seen as a complement to the other available products.

### Downscaling to the daily time scale

The currently available TAMSAT dekadal (v2.0) and pentadal (v3.0) rainfall estimates are disaggregated to daily values in proportion to the amount of CCD observed for each day (each daily CCD map is created by considering all TIR images from 06:00 to 06:00 the following day, to coincide with the timing gauge observations are usually taken). This has the advantage that the estimates are constrained to match the dekadal or pentadal rainfall totals which are expected to be reliable. The daily rainfall estimates are thus calculated according to equation (2):

(2)raindaily=raintimestepCCDtimestep×CCDdaily

where *rain*_*daily*_ is the daily rainfall estimate, *rain*_*timestep*_ is the dekadal (v2.0) or pentadal (v3.0) rainfall estimate, *CCD*_*timestep *_is the CCD summed over the ten or five days and *CCD*_*daily*_ is the daily CCD. The complete process used to create the TAMSAT daily rainfall estimates is illustrated in Fig. 1.

## Data Records

### Data archive

A time-series of daily totals has been generated from 1983 to the present for all of Africa. A day is considered missing if there is a gap of more than six continuous hours in the TIR imagery. For version 2.0, a dekad is considered missing if there are more than two missing days (see Maidment *et al.*^18^ for details), whereas for version 3.0, the pentad is considered missing if more than one day is missing. Despite many incomplete or missing TIR images during the 1980s and early 1990s, both version 2.0 and 3.0 have near-complete archives. For version 2.0 for example, based on available data from the EUMETSAT archive, the dataset is approximately 97% complete (as of December 31st 2016). Specifically, of the 12,419 days between January 1st 1983 and December 31st 2016, there are 398 missing days. Of these, in 271 cases, the whole dekad was missing, resulting in no data to disaggregate, and in 127 cases, individual days within the dekad were missing. Of the missing days, 271 were between 1983 and 1989, 114 were between 1990 and 1999, and 13 were after 2000. There have been no missing days since 2007. As expected, the proportion of missing days is similar for TAMSAT-3. The daily estimates are available from January 11th 1983 to the present and are available within two days after the end of each dekad (i.e., 11th, 21st, and 1st of the following month) for version 2.0 and each pentad (i.e., 6th, 11th, 16th, 21st, 26th and 1st of the following month) for version 3.0.

### Data access and format

The daily rainfall estimates (in mm per day) are freely available as netCDF files for each day from the TAMSAT website (http://www.tamsat.org.uk) and the University of Reading Research Data Archive (version 2.0, Data Citation 1; version 3.0: Data Citation 2). TAMSAT-2 is also available on the International Research Institute for Climate and Society (IRI) Data Library (https://iridl.ldeo.columbia.edu/SOURCES/.Reading/.Meteorology/.TAMSAT/.TARCAT/.v2p0/.daily/), with TAMSAT-3 expected to be available during 2017. The spatial resolution is 0.0375° latitude by 0.0375° longitude with estimates provided for all land points in Africa, including Madagascar. In addition, the TAMSAT website contains quicklook images for each day and a time series extraction tool can be used to extract area-average data for countries, administrative districts and user defined rectangular regions or user defined pixels in csv format. The IRI Data Library includes additional subsetting and data analysis tools.

## Technical Validation

### Study regions and validation data

**Figure 2:**
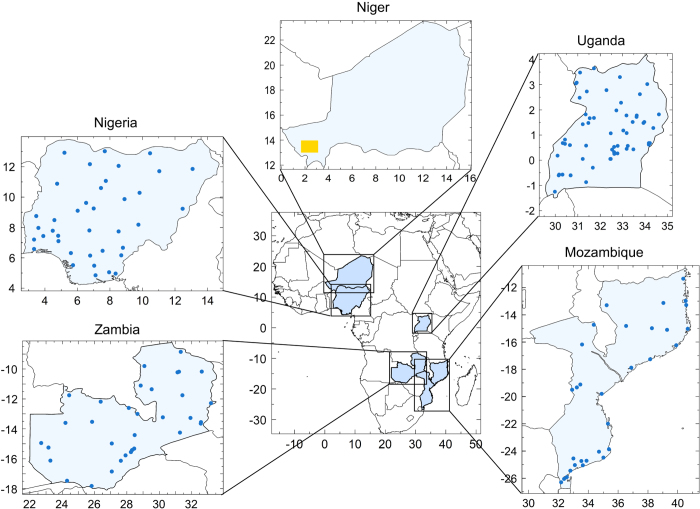
Location of the five rain gauge networks used in this study. The yellow shading over south-west Niger denotes the region covered by the Hapex-Sahel gauge network.

The daily satellite rainfall estimates have been evaluated using rain gauge records covering four countries (Mozambique, Nigeria, Uganda and Zambia) and one region over south-west Niger consisting of a dense network (see Fig. 2 and Table 1). These regions of Africa are characterised by contrasting rainfall climates and thus, the validation provides a useful indicator of the expected skill of the TAMSAT daily estimates (and the other satellite estimates used in this study) across Africa. The section below summarises the general climate of each region considered.

The rainfall over Niger is typical of that experienced over most of the Sahel, characterized by a single rainy season occurring during boreal summer. The main features of the rainy season are the West African Monsoon, which advects moisture-laden air onto the continent and African Easterly Waves that are associated with the passage of westward propagating mesoscale convective systems that are responsible for the majority of rainfall over this part of Africa^35,36^. TIR-based estimation algorithms, including TAMSAT, have demonstrated high skill over the Sahel^9,12,32^. Much of Nigeria’s rainfall climate is similar in nature, although rainfall in the coastal regions and areas surrounding the Cameroon Highlands to the east are often modulated by oceanic and orographic effects respectively, complicating the relationship between cloud top temperature and rainfall^31^.

Most of Uganda experiences two rainy seasons associated with the seasonal northward and southward migration of the Inter-Tropical Convergence Zone^14^. Whilst rainfall is convective in origin, the presence of mountain chains to the east and southwest of the country and large bodies of water, such as Lake Victoria and Lake Albert, influence the local climate considerably. While this presents a challenge for TIR-based algorithms due to the increased occurrence of rainfall from warm clouds, particularly where local changes to the rainfall processes are pronounced, 10-day total satellite-derived estimates have shown to be skilful over this region^14^.

Zambia has one rainy season occurring between October and April. As the country is relatively flat and landlocked and rainfall is primarily a result of convective systems, cold cloud tops of these convective systems and rainfall are usually well correlated, as found across Niger.

Finally, the climate of Mozambique contrasts with the other regions considered in this study. The close proximity to the Indian Ocean and the passage of tropical depressions and cyclones create a varied and complex climate. Such variable weather regimes presents a challenge for TIR-based algorithms, especially when other data (e.g., gauge data) are not incorporated contemporaneously^17^.

The daily rain gauge records from Nigeria, Uganda and Zambia were obtained directly from their respective meteorological agencies. Each of these datasets were subject to rigorous quality control measures. These procedures involved checking for erroneous entries, duplicates, and outliers. If outliers were flagged, temporal and spatial checks were then conducted. The high density Niger dataset was created during the Hydrology-Atmosphere Pilot Experiment in the Sahel (Hapex-Sahel) experiment in the early 1990s (refs 37–40) and has been used in many subsequent studies^10^. Finally, the Mozambique data was sourced from The Mozambique National Institute of Meteorology and quality controlled for The World Bank^41^. Only those records during each region’s rainy season were used (for Uganda, records covering the ‘long rains’ were used). Whilst not all stations used have complete records, each regional dataset had at least 15,000 gauge records available for validation (see Table 1).

The variability in the TAMSAT daily rainfall estimates is derived entirely from the satellite imagery—with the calibration carried out on 10-daily (v2.0) or 5-daily (v3.0) accumulated rainfall/CCD over regions encompassing hundreds of gauge-CCD pairs^8,18^. The evaluation of the TAMSAT rainfall estimates described here can thus arguably be considered to be against independent data, even though some of the gauge records may have been included in the historical calibration. This is not the case for some of the comparison satellite datasets used in this study, which incorporate contemporaneous gauge records. The Niger gauge dataset however, is not included either in the TAMSAT version 2.0 dekadal calibration, or—to our knowledge—in the comparison satellite datasets.

To ensure a consistent comparison between the satellite estimates and ground-based data, all rain gauge records were interpolated onto a regular 0.25° by 0.25° grid using block kriging. Kriging was chosen as it has been shown to be superior compared to other forms of spatial interpolation^42–44^. Since the uncertainty in the interpolated rainfall amount increases significantly away from a rain gauge, only those grid squares containing at least one gauge were used. For simplicity, it was assumed that all 0.25° grid squares containing only dry gauges were set to zero rainfall. In the event of a grid square containing dry and wet gauges, the kriged rainfall amount was used.

It should be noted that given the high density of the Niger gauge network, the interpolated area-average values will, in general, be much more accurate than the equivalent interpolated grid values over the four other regions whose gauge networks are considerably less dense. Moreover, since the availability of the satellite estimates and gauge data for each region do not cover the same time periods, it is not possible to directly compare the results from one region to another. This is particularly the case for Niger whose gauge data is only available for one year. However, the results presented provide a useful indicator of the expected skill of the TAMSAT daily rainfall estimates, in comparison to the other satellite datasets.

The TAMSAT-2 and TAMSAT-3 rainfall estimates were evaluated alongside six other satellite precipitation datasets providing daily estimates. These datasets are CHIRPS, CHIRP (CHIRPS without stations), ARC, NOAA’s African Rainfall Estimates version 2 (hereinafter RFE), the Tropical Rainfall Measuring Mission (TRMM) Multi-satellite Precipitation Analysis (TMPA)-3B42 and NOAA’s Climate Prediction Center (CPC) morphing technique (CMORPH) (see Table 2). The latter three datasets include passive microwave (PMW) imagery, and hence are expected to be capable of providing more realistic information on rainfall intensity. A brief description of these datasets is as follows:

CHIRPS provides 30+ years of high resolution (0.05° lat-lon grid) quasi-global (50°S-50°N and 180°W–180°E) rainfall estimates at daily, pentadal, and monthly time-steps. CHIRPS depends on several data sources to produce estimates of rainfall. First, TIR imagery are used to produce maps of pentadal CCD. Unlike TAMSAT, which implements a temporally and spatially varying threshold to compute the CCD, a constant rain/no-rain threshold of 235 K is used. Calibration regression coefficients are then derived by comparing TMPA-3B42 rainfall estimates (2000–2013) and CCD. These calibration parameters are then applied to the complete CCD record to produce a time-series of rainfall estimates. Next, these pentadal rainfall estimates are expressed as a fraction of their long-term mean (1981–2013) and then multiplied by the Climate Hazards group Precipitation climatology (CHPclim). This step produces what is known as CHIRP, i.e., the satellite-based estimates with no merging of rain gauge records, and is also evaluated in this study. CHPclim is an attempt to create accurate pentadal and monthly climatologies based on rain gauge records and multiple satellite-based products^45^. Finally, station rain gauge records are merged with CHIRP using a modified form of the inverse distance weighting algorithm to create the CHIRPS product. A preliminary version, CHIRPS-prelim, is created with a 2-day latency based on GTS data, while the final version (evaluated in this paper) makes use of public monthly gauge summaries and additional data from meteorological agencies. Daily estimates of precipitation are created by disaggregating the pentadal estimates using daily CCD observations (analogous to the method described in this paper).

Both ARC and RFE produce daily rainfall estimates solely for Africa and were created to aid drought monitoring across sub-Saharan Africa. RFE uses satellite imagery from two streams, namely (1) TIR imagery to create rainfall estimates based on the GOES Precipitation Index (GPI) algorithm^46^ and (2) PMW imagery from the AMSU and SSM/I satellite instruments are used to create rainfall estimates using the method described by Ferraro and Marks^47^. The TIR and PMW rainfall estimates are then merged, before being adjusted to available GTS station data. ARC is a long term (30+ years) dataset and employs a similar method to RFE in that satellite estimates are merged with GTS gauge data, however PMW data are not considered.

The primary objective of the TRMM satellite and the derived products was aimed at improving observations of tropical precipitation^48,49^. The TRMM satellite, equipped with a precipitation radar, as well as microwave imager and a visible-infrared scanner, was used to better estimate precipitation features such as intensity, distribution, and type. 3-hourly TMPA-3B42 (evaluated in this study) estimates are derived from merged-TIR imagery from geostationary and polar-orbiting platforms, adjusted by information derived from the TRMM instruments. The final step used the monthly Global Precipitation Climatology Centre (GPCC) gauge analysis to scale the monthly TMPA estimates to the gauge values. Sub-monthly products, including the 3-hourly TMPA-3B42 estimates, take account of this gauge scaling. TMPA data were issued to provide near-global coverage at a spatial resolution of 0.25°.

CMORPH^50^ produces global rainfall estimates from various PMW sensors. Motion vectors are calculated using half-hourly geostationary TIR imagery, which are used to propagate the PMW precipitation fields forward and back in time where no direct PMW data are available. A time-weighted interpolation is applied to the available PMW estimates to provide an estimate of the rainfall distribution and intensity for the intervening missing half-hour periods. This process is referred to as ‘morphing’ of the observations. For this study, the 3-hourly estimates at a spatial resolution of 0.25° were used.

In the case of CHIRPS, the operational product CHIRPS-Prelim was not considered because at the time this study was conducted, the data were not available prior to 2015. All of the other datasets can be considered fully operational, except TMPA-3B42 which was replaced by the Integrated Multi-satellitE Retrievals for Global Precipitation Measurement (GPM) (IMERG) estimates in 2014. For consistency, all satellite datasets (except TMPA-3B42 and CMORPH) were bilinearly interpolated to a regular grid spacing of 0.25° by 0.25°—same as the kriged gauge grid, and grid squares with coincident gauge measurements were then extracted. When summing the CMORPH and TMPA-3B42 3-hourly estimates to daily totals, the 3-hourly slots corresponding to the TAMSAT day (i.e., 06:00–06:00 the following day) were chosen. Evaluations were then carried out for the period of the gauge data, which differs from region to region (see Table 1).

### Statistical comparison of TAMSAT daily rainfall estimates with rain gauge data and other satellite-based rainfall datasets

The TAMSAT version 2.0 dekadal and monthly estimates and their representation of the Africa-wide climatology and seasonal cycle have been evaluated elsewhere^18^ and hence these features are not assessed here. Similar analyses for TAMSAT version 3.0 are documented on the TAMSAT website. Instead, the paper focuses on the ability of TAMSAT to capture daily rainfall characteristics, i.e., occurrence and amount.

#### Rainfall occurrence

Rainfall occurrence was evaluated using a suite of binary skill scores that encapsulate information on rainy/dry days in a contingency table (see Table 3).

A contingency table has been constructed for each region using all available data and is used to compute the following statistics:

- Accuracy; defined as the fraction of rainfall estimates that were estimated correctly: *(A+D)/(A+B+C+D)*
- Frequency bias (bias); defined as the rainfall estimate frequency of rainy days compared to the gauge observed frequency of rainy days: *(A+B)/(A+C)*
- Probability of detection (POD); defined as the fraction of rainy days correctly estimated: *A/(A+C)*
- False alarm ratio (FAR): defined as the proportion of estimated rainy days that did not actually occur: *B/(A+B)*
- Probability of false detection (POFD); defined as the fraction of gauge observed dry days incorrectly estimated as a rainy day: B/(B+D)
- Equitable threat score (ETS); defined as the fraction of gauge observed rainy days that were correctly estimated allowing for hits due to chance: *(A-A*_*random*_*)/(A+B+C-A*_*random*_) where *A*_*random*_*=(A+C)(A+B)/(A+B+C+D)*
- Peirce’s Skill Score (PSS, also known as Hanssen and Kuipers discriminant); defined as the ability of the satellite estimate to differentiate between a rainy day and a dry day (as given by the gauge observation): (A/(A+C))-(B/(B+D))

**Figure 3:**
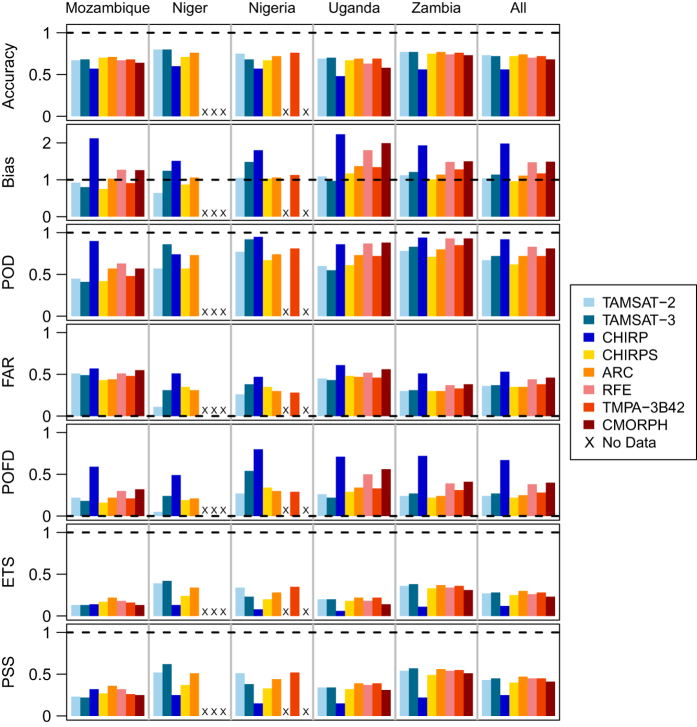
Skill scores for each product and country computed using a threshold of 0 mm (rain gauge and satellite estimate) using all available gauge observations (see Table 1). Horizontal black dashed line denotes the perfect skill score. Blue-shaded colour bars denote those datasets that are considered TIR-only, yellow/orange-shaded colour bars denote those that are merged TIR-gauge and pink/red-shaded colour bars denote PMW-based datasets.

Figure 3 displays barplots for each binary skill score over each of the study regions and over all regions for TAMSAT-2, TAMSAT-3 and the other six satellite datasets (values are also given in Table 4). In general, the TAMSAT skill scores (both versions) are similar to most of the other satellite products on all skill measures. Across all regions (leftmost column in Fig. 3), the accuracy skill measure indicates around 70% of the estimates were correct (i.e., in estimating dry and rainy days) and that around 70–80% of the observed rainy days were captured (POD). However, around 35–45% of estimated rainy days were falsely estimated (FAR) resulting in all products overestimating the occurrence of rainy days (bias), with the errors most severe in CHIRP, RFE and CMORPH. Similarly, around 20–40% of the gauge observed dry days, were estimated as rainy days (POFD). Of the eight datasets, the TIR-based products show more commonality than the PMW-based products. The similarity between skill scores of the former suggests that this is a result of the use of TIR imagery being used to define those regions which are rainy. The exception to these findings is CHIRP, which, across all countries, grossly overestimates the frequency of rainfall events, leading to a high frequency bias (1.98) and POFD (0.67). However, CHIRPS demonstrates marked improvement on all statistical measures compared to CHIRP.

Regionally however, there are some differences. Across most of the satellite products, scores are generally better for Niger, particularly for TAMSAT-2, which has the best scores for accuracy, FAR and POFD, and TAMSAT-3, which has the best scores for accuracy (same value as TAMSAT-2), POD, ETS and PSS. Scores are generally worst for Mozambique and Uganda. This is consistent with the expectation that satellite rainfall estimation algorithms, even those that incorporate PMW imagery, generally perform worse when the rainfall climate is strongly modulated by large water bodies, and for regions in close proximity to the ocean and complex topography^17^. Conversely, such algorithms perform well in the Sahel and over Zambia, where rainfall is primarily convective and the rainfall climate is less variable spatially. The high skill across both Niger and Zambia reflects this.

The skill scores were also assessed as a function of rainfall threshold (i.e., changing the satellite rainfall estimate threshold at which the contingency table is constructed). However, for all datasets the skill scores exhibited no improvement in skill as the threshold was increased from 0 mm up to 40 mm (not shown).

#### Rainfall amount

**Figure 4:**
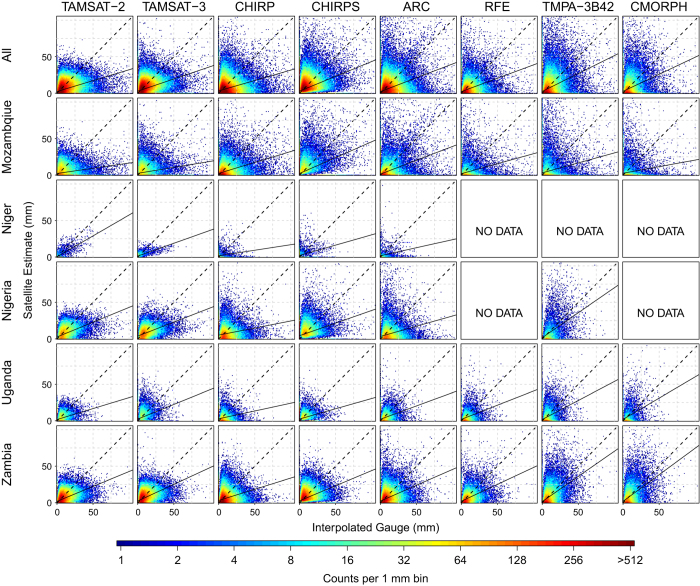
Scatterplot of daily satellite rainfall estimates against kriged gauge estimates for 0.25° grid cells that contain at least one rain gauge. Scale gives the counts per 1 mm bin. Dashed line indicates the one-to-one correspondence; solid line gives the linear regression best fit.

**Figure 5:**
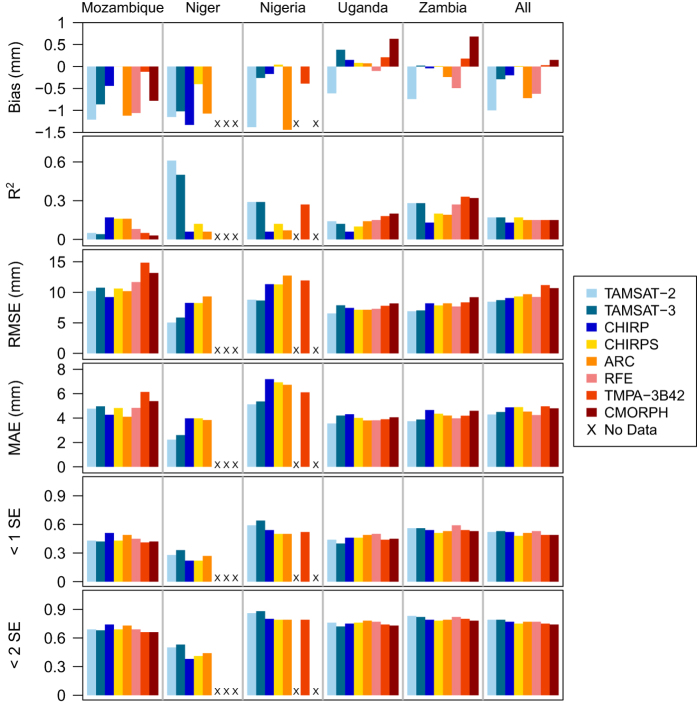
Same as Fig. 3, but for rainfall amount skill measures.

Figure 4 shows a density scatterplot of TAMSAT-2, TAMSAT-3 and the other satellite rainfall estimates against kriged rain gauge amounts for all regions included in this study. Quantitative assessment of rainfall amount was based on the calculation of bias, coefficient of determination (R^2^), root mean square error (RMSE), and mean absolute error (MAE). The kriging process also generates an estimate of the uncertainty of the interpolated gauge grid value. Using this, the fraction of satellite estimates within one and two standard errors of the gauge value was also computed. A summary of the aforementioned statistics is given in Fig. 5 for each dataset and for each region (values are also given in Table 5).

**Figure 6:**
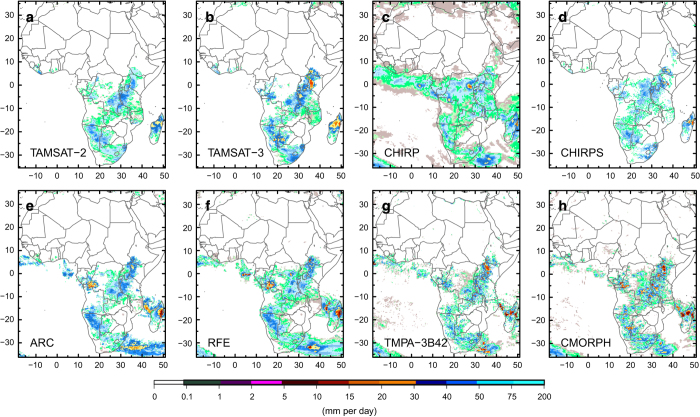
Africa-wide rainfall fields (at 0.25° by 0.25° resolution) for January 1st 2010 from each daily satellite-based dataset. (**a**) TAMSAT-2, (**b**) TAMSAT-3, (**c**) CHIRP, (**d**) CHIRPS, (**e**) ARC, (**f**) RFE, (**g**) TMPA-3B42 and (**h**) CMORPH.

There is some correlation between rainfall estimates and gauge measured rainfall amount for all of the satellite-rainfall estimation datasets, but there are also significant discrepancies (see Fig. 4). For example, TAMSAT-2 systematically underestimates rainfall amount, and does not distinguish between moderate and high rainfall. Figure 5 confirms that there is a negative TAMSAT-2 bias for all countries, with the largest bias being for Mozambique and Nigeria. The correlations (i.e., R^2^) between gauge and TAMSAT-2 rainfall amounts range from 0.05 (Mozambique) to 0.61 (Niger). Niger also has the lowest errors (RMSE and MAE) out of the five regions whereas Mozambique has the largest RMSE. TAMSAT-3 however demonstrates improvement on some on the statistics considered when compared to TAMSAT-2, most notably, a reduction in the dry bias. There is also slightly better distinction between moderate and high rainfall (c.f. Figs 4 and 6).

When the TAMSAT estimates are contrasted with the other rainfall datasets, it can be seen that over all countries, TAMSAT is in general, comparable in all skill measures, except for bias. CHIRPS has the smallest bias, which can be attributed to the bias removal procedure implemented in the rainfall estimation approach. TAMSAT-2 and TAMSAT-3 estimates typically have smaller errors, as given by lower RMSE and MAE values. The smallest errors are for Niger. Low R^2^ values (with the exception of TAMSAT over Niger) indicate limited skill in representing variability for all datasets. Given the high density gauge network over Niger and the contiguous 0.25° grid squares used here, measures of variability (i.e., R^2^) are associated with both spatial and temporal variability. All datasets typically perform worse over Mozambique as evident by the large spread of data points in Fig. 4. Despite including PMW data in their estimation approaches, neither RFE, TMPA-3B42 and CMOPRH demonstrate substantial improvements in skill over the TIR-based methods, particularly for rainfall amount variability. This indicates that at such fine scales (daily and 0.25°), no dataset considered here can provide robust estimates of daily rainfall amount. This is in agreement with other studies at such scales^31,33,51^.

For all of the regions other than Niger, it is likely that at least some of the validation gauge records have been ingested into the rainfall estimation process for ARC, RFE and CHIRPS. While the high gauge density may be a factor, it is notable that TAMSAT has significantly more skill than the other datasets for Niger, in particular, the relatively high R^2^ values for both TAMSAT-2 and TAMSAT-3. As TAMSAT is the only dataset considered here that is locally calibrated for both rainfall occurrence and rainfall amount, the skill of the TAMSAT data is noteworthy given it does not include contemporaneous information from gauges or PMW imagery. This illustrates the importance and the utility of a local and historical calibration approach.

Figure 6 gives an example of rainfall estimates for January 1st 2010. It can be seen that while the rainfall fields have similar spatial structures, there are fewer intensely rainy pixels in TAMSAT-2 (compared to the other datasets), although this is ameliorated somewhat in TAMSAT-3. While the rainy areas are similar for all of the datasets, the intensities vary considerably. This is consistent with the quantitative analysis described in this study, which showed that for all of the datasets, occurrence is more reliably estimated than amount across the five countries considered.

## Usage Notes

The TAMSAT system was originally designed for seasonal early warning of drought. Until the initial release of daily TAMSAT-2 in 2012, ARC in 2013, and CHIRPS in 2014, long-term satellite-based rainfall data for drought early warning have typically been released at the dekadal time scale. This paper has presented the daily version of the TAMSAT data (versions 2.0 and 3.0). TAMSAT has previously been demonstrated to have good skill for 10-day cumulative rainfall estimates^14^ and we have shown here that the daily data reliably represents the occurrence of rainfall, capturing, on average, around 70 % of observed rainy days (POD) and falsely estimating less than 40 % of rainy days (FAR) across the case study countries. Regionally, TAMSAT captured rainy and non-rainy days better across Niger and Zambia—regions whose rainfall climates are not significantly modulated by large water bodies and complex topography. Variability in rainfall amount is, however, not well captured. Whilst the ability to differentiate between low and high rainfall amounts is important, it can be argued that across Africa, long dry spells (which, to be detected, require satellite estimation algorithms to skilfully differentiate a rainy day from a non-rainy day) is more damaging to crops than extremes of rainfall^1^. Many aspects of the skill of the TAMSAT daily data are however similar or better (depending on the skill measure) than other, widely used African operational daily datasets. Since CHIRPS, ARC, and RFE make use of contemporaneous gauges which are likely included in the validation datasets, this complicates the interpretation of the results.

An obvious application for the daily data is the production of rainfall estimates for periods other than 5-day or 10-day accumulations starting on fixed days of the calendar month. The availability of a daily version of the TAMSAT dataset gives a choice of products based on the optimal length and starting point of cumulative rainfall estimates required. This facilitates comparison with other datasets, which are issued at weekly resolutions for example, and allows for greater flexibility for agricultural and hydrological applications.

Many crop and hydrological models require daily input^52–55^. In the case of crop modelling, yield generally depends on cumulative rainfall for key parts of the growing cycle. Daily data are therefore useful because the data can pick out key development phases of crops and is an example of the value of being able to cumulate rainfall over bespoke periods. Although TAMSAT data may be too coarse for analysis of small catchments, hydrological models for medium and large catchments may be able to utilise data at 4-km resolution^56^. The TAMSAT data have most skill when spatially aggregated^4,14^, and this is especially the case for rainfall that is not aggregated in time. In this context, the suitability of TAMSAT daily rainfall estimates depends on the hydrological features of the catchment and the purpose of the monitoring or modelling. TAMSAT’s poor skill for rainfall amount means that it is most suitable for monitoring large catchments where river discharge is determined by gradual accumulation of rainfall over a period of days. It can be argued that the TAMSAT data is not suitable for providing information on pluvial flood risk.

Unlike the other daily rainfall datasets considered, TAMSAT does not incorporate gauge data in real time. Recent studies have shown that inconsistencies in the gauge record can lead to spurious trends in rainfall, especially in the tropics, where the station network is patchy^21,57^. The TAMSAT cumulative rainfall datasets and the derived daily estimates can therefore be considered temporally consistent, which is important in both assessing climatic risks and for seasonal rainfall monitoring. As such, TAMSAT daily data are well suited to the study of long term changes in daily metrics, relating primarily to occurrence, such as the length of dry spells and the length of the growing season^58^. Since it cannot capture the intensity of high rainfall events well, TAMSAT daily data is less suited for studies of long term changes in rainfall amount.

In conclusion, we present the TAMSAT high-resolution daily rainfall dataset for Africa. The data are back calculated to January 1983 and updated in near-real time (v2.0 is updated every ten days and v3.0 is updated every five days). The recent development of TAMSAT version 3.0 pentadal estimates and derived daily estimates removes spatial artefacts and greatly reduces the dry bias associated with the previous version. A formal statistical assessment indicates that both TAMSAT daily datasets have comparable skill to other remotely sensed rainfall datasets, and can therefore be used for similar applications. Furthermore, TAMSAT’s historical calibration suits it well for risk assessment and the investigation of long-term changes in the rainfall climate.

## Additional Information

**How to cite this article:** Maidment, R. I. *et al.* A new, long-term daily satellite-based rainfall dataset for operational monitoring in Africa. *Sci. Data* 4:170063 doi: 10.1038/sdata.2017.63 (2017).

**Publisher’s note:** Springer Nature remains neutral with regard to jurisdictional claims in published maps and institutional affiliations.

## Supplementary Material



## Figures and Tables

**Table 1:** Summary of the rain gauge data used in the validation of the daily rainfall estimates.

**Country**	**No. of Stations**	**Years**	**Months**	**No. of Records**	**Station data coverage**			
					**<50%**	**50–75%**	**75–90%**	**>90%**
Mozambique	31	1983–2009	Oct-Apr	153,387	2	4	6	19
Niger	107	1992	May-Sep	15,600	0	9	9	89
Nigeria	38	1991–2000	Mar-Oct	86,951	0	1	10	27
Uganda	56	2000–2005	Feb-Jun	34,864	18	8	10	20
Zambia	33	1983–2013	Oct-Apr	192,873	1	4	6	22

**Table 2:** Summary of the daily satellite rainfall datasets used in this study.

**Product**	**Spatial resolution**	**Starting year**	Inputs*	**Latency**	**Reference**
TAMSAT v2.0	0.0375°	1983	TIR, gauge	Up to 12 days	—
TAMSAT v3.0	0.0375°	1983	TIR, gauge	Up to 7 days	—
CHIRP	0.05°	1981	TIR, TMPA-3B42	Up to 7 days	Funk *et al.*^20,45^
CHIRPS v2.0	0.05°	1981	TIR, TMPA-3B42, gauge	Up to 7 weeks	Funk *et al.*^20,45^
ARC v2.0	0.1°	1983	TIR, gauge	1 day	Novella and Thiaw^19^
RFE v2.0	0.1°	2001	TIR, PMW, gauge	1 day	http://www.cpc.ncep.noaa.gov/products/fews/RFE2.0_tech.pdf
TMPA-3B42 v7.0	0.25°	1997	TIR, PMW, radar, gauge	No longer operational	Huffman *et al.*^49^
CMORPH	0.25°	2002	TIR, PMW	18 h	Joyce *et al.*^50^

*PMW=passive microwave; TIR=thermal infra-red.

**Table 3:** Contingency table used for the statistical analysis of rainfall occurrence for the daily satellite rainfall estimates.

		Gauge Observations	
		Rain	No Rain
**Satellite Rainfall Estimate**	**Rain**	Hits 'A'	False Alarms 'B'
	**No Rain**	Misses 'C'	Correct Negatives 'D'

**Table 4:** Skill scores computed using a threshold of 0 mm for each country (the threshold is defined as the rainfall amount that distinguishes a dry day from a rainy day).

**Country**	**Dataset**	***N***	**Accuracy**	**Bias**	**POD**	**FAR**	**POFD**	**ETS**	**PSS**
Mozambique	TAMSAT-2	147,059	0.67	0.92	0.45	0.51	0.22	0.13	0.23
	TAMSAT-3	123,586	0.68	0.80	0.41	0.49	0.18	0.13	0.22
	CHIRP	153,078	0.57	2.12	**0.90**	0.57	0.59	0.14	0.32
	CHIRPS	114,204	0.70	0.75	0.42	**0.43**	**0.16**	0.17	0.27
	ARC	145,600	**0.71**	**1.03**	0.57	0.44	0.22	**0.22**	**0.36**
	RFE	49,935	0.67	1.27	0.63	0.51	0.30	0.18	0.32
	TMPA-3B42	65,719	0.68	0.91	0.48	0.48	0.21	0.16	0.26
	CMORPH	37,813	0.64	1.26	0.57	0.55	0.32	0.13	0.25
Niger	TAMSAT-2	3,671	**0.80**	0.64	0.57	**0.11**	**0.05**	0.39	0.52
	TAMSAT-3	3,671	**0.80**	1.24	**0.86**	0.31	0.24	**0.42**	**0.62**
	CHIRP	3,671	0.60	1.51	0.74	0.51	0.49	0.13	0.25
	CHIRPS	3,671	0.71	0.87	0.57	0.35	0.19	0.24	0.37
	ARC	3,623	0.76	**1.06**	0.73	0.31	0.21	0.34	0.51
	RFE	—	—	—	—	—	—	—	—
	TMPA-3B42	—	—	—	—	—	—	—	—
	CMORPH	—	—	—	—	—	—	—	—
Nigeria	TAMSAT-2	64,527	0.75	1.05	0.77	**0.26**	**0.27**	0.34	0.51
	TAMSAT-3	63,257	0.68	1.48	0.92	0.38	0.54	0.23	0.38
	CHIRP	65,290	0.57	1.80	**0.95**	0.47	0.80	0.08	0.15
	CHIRPS	65,290	0.67	**1.03**	0.67	0.35	0.34	0.02	0.33
	ARC	64,381	0.72	1.06	0.74	0.30	0.30	0.28	0.44
	RFE	—	—	—	—	—	—	—	—
	TMPA-3B42	17,815	**0.76**	1.13	0.81	0.28	0.29	**0.35**	**0.52**
	CMORPH	—	—	—	—	—	—	—	—
Uganda	TAMSAT-2	31,084	0.69	1.09	0.60	0.45	0.26	0.20	0.34
	TAMSAT-3	30,949	**0.70**	**0.97**	0.55	**0.43**	**0.22**	0.20	0.34
	CHIRP	31,084	0.48	2.23	0.86	0.61	0.71	0.06	0.15
	CHIRPS	31,084	0.67	1.17	0.61	0.48	0.29	0.18	0.32
	ARC	31,020	0.69	1.37	0.73	0.47	0.34	**0.22**	**0.39**
	RFE	25,207	0.63	1.80	0.87	0.52	0.50	0.18	0.37
	TMPA-3B42	30,975	0.69	1.34	0.72	0.46	0.33	**0.22**	**0.39**
	CMORPH	14,082	0.58	1.99	**0.88**	0.56	0.56	0.14	0.31
Zambia	TAMSAT-2	169,021	**0.77**	1.12	0.78	**0.30**	0.24	0.36	0.54
	TAMSAT-3	161,074	**0.77**	1.21	0.83	0.31	0.27	0.38	0.57
	CHIRP	175,080	0.56	1.93	**0.94**	0.51	0.72	**0.11**	**0.22**
	CHIRPS	175,080	0.75	**1.01**	0.71	**0.30**	**0.22**	0.33	0.49
	ARC	167,530	**0.77**	1.14	0.80	**0.30**	0.24	0.37	0.56
	RFE	65,538	0.74	1.48	0.93	0.37	0.39	0.34	0.54
	TMPA-3B42	83,560	0.76	1.28	0.85	0.33	0.31	0.36	0.55
	CMORPH	53,458	0.73	1.50	0.93	0.38	0.41	0.31	0.51
All	TAMSAT-2	415,362	0.73	**1.04**	0.67	0.36	0.24	0.27	0.43
	TAMSAT-3	382,537	0.72	1.14	0.72	0.37	0.27	0.28	0.45
	CHIRP	428,203	0.56	1.98	**0.92**	0.53	0.67	0.12	0.25
	CHIRPS	389,329	0.72	**0.96**	0.62	**0.35**	**0.22**	0.25	0.40
	ARC	412,154	**0.74**	1.11	0.72	**0.35**	0.25	**0.30**	**0.47**
	RFE	140,680	0.70	1.47	0.83	0.44	0.38	0.26	0.45
	TMPA-3B42	198,069	0.72	1.17	0.72	0.38	0.28	0.28	0.45
	CMORPH	105,353	0.68	1.49	0.81	0.46	0.40	0.23	0.41
*N* is the number of coincident gauge-satellite grid observations used. Values in bold denote the most favourable comparison.									

**Table 5:** Comparison of satellite rainfall estimates and collocated kriged gauge records for each country and for each satellite product.

**Country**	**Dataset**	***N***	**Bias (mm)**	**R**^**2**^	**RMSE (mm)**	**MAE (mm)**	**<1 s.e.**	**<2 s.e.**
Mozambique	TAMSAT-2	147,059	−1.21	0.05	10.20	4.76	0.43	0.69
	TAMSAT-3	123,586	−0.86	0.04	10.75	4.96	0.42	0.68
	CHIRP	153,078	−0.44	**0.17**	**9.21**	4.27	**0.51**	**0.74**
	CHIRPS	114,204	**0.00**	0.16	10.61	4.82	0.43	0.69
	ARC	145,600	−1.12	0.16	10.19	**4.10**	0.49	0.73
	RFE	49,935	−1.06	0.08	11.67	4.83	0.45	0.69
	TMPA-3B42	65,719	−0.12	0.05	14.85	6.14	0.41	0.66
	CMORPH	37,813	−0.78	0.03	13.17	5.38	0.42	0.66
Niger	TAMSAT-2	3,671	−1.15	**0.61**	**5.04**	**2.22**	0.28	0.50
	TAMSAT-3	3,671	−1.02	0.50	5.85	2.60	**0.33**	**0.53**
	CHIRP	3,671	−1.33	0.06	8.27	3.97	0.22	0.38
	CHIRPS	3,671	**−0.40**	0.12	8.24	3.98	0.22	0.41
	ARC	3,623	−1.07	0.06	9.31	3.83	0.27	0.44
	RFE	—	—	—	—	—	—	—
	TMPA-3B42	—	—	—	—	—	—	—
	CMORPH	—	—	—	—	—	—	—
Nigeria	TAMSAT-2	64,527	−1.38	**0.29**	8.77	**5.12**	0.59	0.86
	TAMSAT-3	63,257	−0.26	**0.29**	**8.65**	5.36	**0.64**	**0.88**
	CHIRP	65,290	−0.17	0.06	11.33	7.19	0.54	0.80
	CHIRPS	65,290	**0.04**	0.12	11.28	6.93	0.50	0.79
	ARC	64,381	−1.44	0.07	12.73	6.72	0.50	0.79
	RFE	—	—	—	—	—	—	—
	TMPA-3B42	17,815	−0.39	0.27	11.93	6.10	0.52	0.79
	CMORPH	—	—	—	—	—	—	—
Uganda	TAMSAT-2	31,084	−0.61	0.14	**6.53**	**3.55**	0.44	0.76
	TAMSAT-3	30,949	0.38	0.12	7.88	4.20	0.40	0.72
	CHIRP	31,084	0.15	0.06	7.43	4.31	0.46	0.75
	CHIRPS	31,084	0.08	0.10	7.13	4.01	0.46	0.76
	ARC	31,020	**0.07**	0.14	7.14	3.80	0.49	**0.78**
	RFE	25,207	−0.10	0.15	7.29	3.81	**0.50**	0.77
	TMPA-3B42	30,975	0.21	0.18	7.78	3.90	0.44	0.74
	CMORPH	14,082	0.63	**0.20**	8.18	4.06	0.45	0.73
Zambia	TAMSAT-2	169,021	−0.74	0.28	**6.89**	**3.74**	0.56	**0.83**
	TAMSAT-3	161,074	0.02	0.28	7.02	3.88	0.56	0.82
	CHIRP	175,080	−0.04	0.13	8.20	4.66	0.54	0.79
	CHIRPS	175,080	**0.01**	0.20	7.85	4.35	0.51	0.78
	ARC	167,530	−0.24	0.19	8.20	4.20	0.53	0.79
	RFE	65,538	−0.49	0.27	7.68	3.97	**0.59**	0.82
	TMPA-3B42	83,560	0.18	**0.33**	8.35	4.19	0.54	0.80
	CMORPH	53,458	0.68	0.32	9.18	4.59	0.53	0.78
All	TAMSAT-2	415,362	−1.00	**0.17**	**8.46**	**4.29**	0.52	**0.79**
	TAMSAT-3	382,537	−0.29	**0.17**	8.71	4.49	**0.53**	**0.79**
	CHIRP	428,203	−0.20	0.13	9.05	4.87	0.52	0.77
	CHIRPS	389,329	**0.01**	**0.17**	9.31	4.89	0.48	0.75
	ARC	412,154	−0.72	0.15	9.69	4.53	0.51	0.77
	RFE	140,680	−0.62	0.15	9.24	4.25	**0.53**	0.77
	TMPA-3B42	198,069	0.03	0.15	11.17	4.96	0.49	0.75
	CMORPH	105,353	0.15	0.15	10.68	4.80	0.49	0.74
*N* is the number of coincident gauge-satellite observations used. Shown are the mean bias, coefficient of determination (R^2^), root mean square error (RMSE), mean absolute error (MAE) and the fraction of the satellite estimates that are within one and two standard errors (s.e.) of the kriged gauge observations. Values in bold denote the most favourable comparison.								
