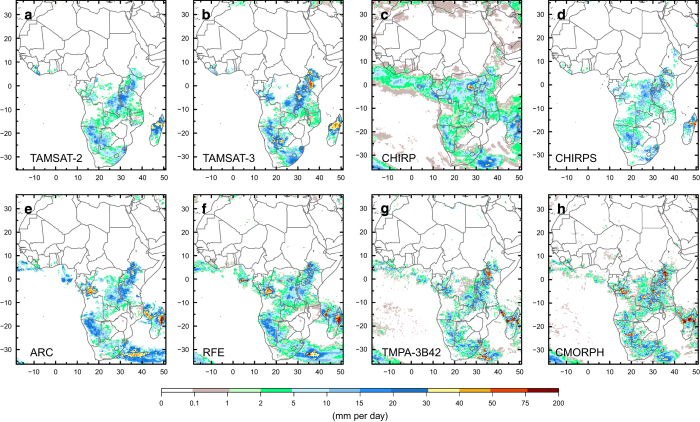# Erratum: A new, long-term daily satellite-based rainfall dataset for operational monitoring in Africa

**DOI:** 10.1038/sdata.2017.82

**Published:** 2017-07-11

**Authors:** Ross I. Maidment, David Grimes, Emily Black, Elena Tarnavsky, Matthew Young, Helen Greatrex, Richard P. Allan, Thorwald Stein, Edson Nkonde, Samuel Senkunda, Edgar Misael Uribe Alcántara

**Keywords:** Natural hazards, Climate sciences, Hydrology

*Scientific Data* 4:170063 doi: 10.1038/sdata.2017.63 (2017); Published 23 May 2017; Updated 11 July 2017

In Fig. 6 of this Data Descriptor the colour gradient of the scale bar was inadvertently modified during the production process. The correct version of Fig. 6 appears below as Fig. 1.

## Figures and Tables

**Figure 1:**